# Supplementary material for: Molecular Epidemiology Reveals the Co-Circulation of Two Genotypes of Coxsackievirus B5 in China
Source: Viruses. 2022 Nov 30;14(12):2693. doi: 10.3390/v14122693 (PMC9785520; doi:10.3390/v14122693)
Supplement: Supplementary file 1 [file viruses-14-02693-s001.zip › viruses-2015730-supplementary.pdf]

Supplement Table S1. The 53 full-length genome sequences of CVB5 used in this study

Year	Isolate province	Name of the sequence	Cases	Specimen type	GneBank number	Genotype
2001	CHN/AnH	CVB5/AnH_44/CHN/2001	AM	N/A	N/A	D
2010	CHN/HuN	CVB5/HuN-81/CHN/2010	HFMD	feces	OP672300	D
2010	CHN/HuN	CVB5/HuN-40/CHN/2010	HFMD	feces	N/A	D
2013	CHN/HB	CVB5/HB-351/CHN/2013	HFMD	rectal swab	OP672301	D
2013	CHN/JX	CVB5/JX-52/CHN/2013	HFMD	rectal swab	N/A	D
2014	CHN/LN	CVB5/LN-202/CHN/2014	HFMD	feces	N/A	D
2014	CHN/JS	CVB5/JS_B006/CHN/2014	HFMD	Nasopharyngeal swab	N/A	D
2014	CHN/JS	CVB5/JS_B007/CHN/2014	HFMD	Nasopharyngeal swab	N/A	D
2014	CHN/JS	CVB5/JS_B015/CHN/2014	HFMD	Nasopharyngeal swab	N/A	D
2014	CHN/JS	CVB5/JS_C014/CHN/2014	HFMD	rectal swab	N/A	D
2014	CHN/JS	CVB5/JS_C045/CHN/2014	HFMD	rectal swab	N/A	D
2014	CHN/JS	CVB5/JS_C157/CHN/2014	HFMD	rectal swab	N/A	D
2014	CHN/JS	CVB5/JS_C294/CHN/2014	HFMD	rectal swab	OP672302	D
2014	CHN/JS	CVB5/JS_C315/CHN/2014	HFMD	rectal swab	N/A	D
2014	CHN/JS	CVB5/JS-202/CHN/2014	HFMD	Nasopharyngeal swab	N/A	D
2014	CHN/JS	CVB5/JS-281/CHN/2014	HFMD	Nasopharyngeal swab	N/A	D
2014	CHN/SaX	CVB5/SaX-539/CHN/2014	HFMD	rectal swab	N/A	D
2014	CHN/SaX	CVB5/SaX-568/CHN/2014	HFMD	rectal swab	N/A	D
2015	CHN/GS	CVB5/GS-514/CHN/2015	HFMD	Feces	OP672303	D
2016	CHN/XJ	CVB5/XJ-74/CHN5/2016	HFMD	feces	OP672304	D
2017	CHN/JS	CVB5/JS-41/CHN/2017	HFMD	Nasopharyngeal swab	OP672305	D
2017	CHN/JL	CVB5/JL-17/CHN/2017	HFMD	N/A	N/A	D
2017	CHN/JL	CVB5/JL-18/CHN/2017	HFMD	N/A	N/A	D
2017	CHN/JL	CVB5/JL-19/CHN/2017	HFMD	N/A	OP672306	D
2017	CHN/JL	CVB5/JL-20/CHN/2017	HFMD	N/A	N/A	D
2017	CHN/JL	CVB5/JL-25/CHN/2017	HFMD	N/A	N/A	D
2017	CHN/XJ	CVB5/XJ-86/CHN/2017	HFMD	feces	OP672307	D
2018	CHN/QH	CVB5/QH-54/CHN/2018	HFMD	Nasopharyngeal swab	OP672308	D
2018	CHN/TJ	CVB5/TJ_112/CHN/2018	HFMD	Nasopharyngeal swab	N/A	D
2018	CHN/TJ	CVB5/TJ-113/CHN/2018	HFMD	Nasopharyngeal swab	N/A	D
2018	CHN/TJ	CVB5/TJ-95/CHN/2018	HFMD	N/A	N/A	D
2018	CHN/TJ	CVB5/TJ-111/CHN/2018	HFMD	Nasopharyngeal swab	N/A	B
2018	CHN/TJ	CVB5/TJ-115/CHN/2018	HFMD	feces	N/A	B
2018	CHN/TJ	CVB5/TJ-114/CHN/2018	HFMD	feces	N/A	B
2018	CHN/TJ	CVB5/TJ-76/CHN/2018	HFMD	N/A	N/A	D
2018	CHN/HLJ	CVB5/HLJ-137/CHN/2018	HFMD	N/A	N/A	B
2018	CHN/HeN	CVB5/HeN-149/CHN/2018	HFMD	feces	N/A	D
2018	CHN/HeN	CVB5/HeN-221/CHN/2018	HFMD	feces	N/A	D
2018	CHN/HeN	CVB5/HeN-293/CHN/2018	HFMD	feces	N/A	D
2018	CHN/HeN	CVB5/HeN-436/CHN/2018	HFMD	Nasopharyngeal swab	N/A	D
2018	CHN/HeN	CVB5/HeN-67/CHN/2018	HFMD	Nasopharyngeal swab	N/A	D
2018	CHN/HeN	CVB5/HeN-69/CHN/2018	HFMD	Nasopharyngeal swab	N/A	D

2018	CHN/GS	CVB5/GS-218/CHN/2018	HFMD	Nasopharyngeal swab	N/A	D
2018	CHN/CQ	CVB5/CQ-43/CHN/2018	HFMD	feces	OP672309	D
2018	CHN/HeN	CVB5/HeN-438/CHN/2018	HFMD	N/A	N/A	D
2018	CHN/HeN	CVB5/HeN-637/CHN/2018	HFMD	Nasopharyngeal swab	N/A	D
2018	CHN/HeN	CVB5/HeN-39/CHN/2018	HFMD	feces	N/A	D
2018	CHN/HeN	CVB5/HeN-199/CHN/2018	HFMD	feces	N/A	D
2018	CHN/HeN	CVB5/HeN-214/CHN/2018	HFMD	feces	N/A	D
2018	CHN/HeN	CVB5/HeN-332/CHN/2018	HFMD	feces	N/A	D
2018	CHN/TJ	CVB5/TJ-76/CHN/2018	HFMD	N/A	N/A	D
2018	CHN/NX	CVB5/NX-1805/CHN/2018	HFMD	Nasopharyngeal swab	N/A	B
2020	CHN/HB	CVB5/HB-056/CHN/2020	HFMD	rectal swab	N/A	B

N/A: indicates unknown.

Supplemental Table S2. Migration paths based on the value of Bayes factor

From	To	Bayes-Factor	Posterior Probability
NC	EC	49.72223255	0.958782358
NC	SC	1.649113726	0.435507166
NC	CC	232.4963	0.990889901
NC	SWC	0.725980128	0.253527386
NC	NEC	0.467387354	0.179424508
NC	NWC	0.141810328	0.062215309
EC	SC	0.354041752	0.142095323
EC	CC	0.167203158	0.072547495
EC	SWC	0.143161293	0.062770803
EC	NEC	0.498436602	0.189090101
EC	NWC	10.0705683	0.824908344
SC	CC	1.374688658	0.391400955
SC	SWC	37.12771697	0.945561604
SC	NEC	0.919338084	0.300744362
SC	NWC	0.380783833	0.151205422
CC	SWC	1.002657525	0.319297856
CC	NEC	0.590376148	0.216420398
CC	NWC	0.108286595	0.048216865
SWC	NEC	1.049999763	0.329407844
SWC	NWC	0.383093311	0.151983113
NEC	NWC	24.43701319	0.919564493

Supplemental Table S3. Whole transmission network based on the location rates

From-To	Location.Rates
location.rates.CC.NC	0.985
location.rates.CC.NEC	1.63

location.rates.CC.NWC	0.942
location.rates.CC.SC	0.918
location.rates.CC.SWC	0.956
location.rates.EC.NC	0.912
location.rates.EC.NEC	0.949
location.rates.EC.NWC	0.96
location.rates.EC.SC	0.866
location.rates.NC.NEC	1.132
location.rates.NC.NWC	1.968
location.rates.NC.SC	0.959
location.rates.NC.SWC	0.91
location.rates.NEC.NWC	1.065
location.rates.NEC.SC	0.972
location.rates.NEC.SWC	0.961
location.rates.NWC.SC	1.003
location.rates.NWC.SWC	0.907
location.rates.SC.SWC	0.937
